# Passively parallel regularized stokeslets

**DOI:** 10.1098/rsta.2019.0528

**Published:** 2020-08-03

**Authors:** Meurig T. Gallagher, David J. Smith

**Affiliations:** 1 Centre for Systems Modelling and Quantitative Biomedicine, University of Birmingham, Birmingham B15 2TT, UK; 2 Institute for Metabolism and Systems Research, University of Birmingham, Birmingham B15 2TT, UK; 3 School of Mathematics, University of Birmingham, Birmingham B15 2TT, UK

**Keywords:** cilia, flagella, GPU, regularized stokeslets

## Abstract

Stokes flow, discussed by G.G. Stokes in 1851, describes many microscopic biological flow phenomena, including cilia-driven transport and flagellar motility; the need to quantify and understand these flows has motivated decades of mathematical and computational research. Regularized stokeslet methods, which have been used and refined over the past 20 years, offer significant advantages in simplicity of implementation, with a recent modification based on nearest-neighbour interpolation providing significant improvements in efficiency and accuracy. Moreover this method can be implemented with the majority of the computation taking place through built-in linear algebra, entailing that state-of-the-art hardware and software developments in the latter, in particular multicore and GPU computing, can be exploited through minimal modifications (‘passive parallelism’) to existing Matlab computer code. Hence, and with widely available GPU hardware, significant improvements in the efficiency of the regularized stokeslet method can be obtained. The approach is demonstrated through computational experiments on three model biological flows: undulatory propulsion of multiple *Caenorhabditis elegans*, simulation of progression and transport by multiple sperm in a geometrically confined region, and left–right symmetry breaking particle transport in the ventral node of the mouse embryo. In general an order-of-magnitude improvement in efficiency is observed. This development further widens the complexity of biological flow systems that are accessible without the need for extensive code development or specialist facilities.

This article is part of the theme issue ‘Stokes at 200 (part 2)’.

## 1. Introduction

Stokes flow describes the fluid mechanics of a vast range of microscopic life, for example the motility and generation of feeding currents by microorganisms, and organ cleansing, gamete/embryo transport and developmental patterning in higher organisms. Typically flow and locomotion involve the action of individual or multiple slender organelles termed flagella and cilia, with the effects of cell surfaces, surrounding cavities and sometimes free surfaces playing crucial roles through hydrodynamic interaction.

This biological relevance has motivated decades of research into what we now refer to as the Stokes flow equations, originally studied (with the inclusion of an unsteady term) by Stokes [1], which describe Newtonian fluid dynamics in the inertialess regime associated with microscopic length scales. The field is too broad to do justice to here, so we mention a few highlights, including Gray & Hancock’s study [2] of the mechanism of sea urchin sperm propulsion and associated slender body theory (ref. [3], see also [4–6]), Chwang & Wu’s work on helical propulsion (ref. [7], see also [8,9]), Blake’s squirmer model [10] and method of images [11] (see also [12–17]), Pedley, Kessler and colleagues’ studies of algal suspension dynamics and gyrotaxis [18,19], the discovery of Stokes flow as the first left–right asymmetric event in vertebrate embryo development [20,21] (see also [22]), Cortez and colleagues’ development of the method of regularized stokeslets [23,24], and Goldstein and colleagues’ discoveries on algal cilia fluid mechanics, from single cell to colony scales [25]. For further review see for example references [26–30].

Numerical techniques, such as the method of regularized stokeslets, have become increasingly valuable in progressing our understanding of biological Stokes flow; in this manuscript we will briefly review this approach, focusing on numerical discretization techniques that achieve a balance between ease-of-implementation and computational efficiency. We will discuss how this method can be extended to use GPU processing capabilities. Algorithms vary greatly in how they improve in performance on parallel processing architectures in general, and GPU hardware in particular. We therefore assess what effect this has on the computational cost of the method by benchmarking against previous work [31,32], as well as providing new simulations of multiple undulatory swimmers and investigating whether sperm-like swimmers are capable of driving particle transport through an enclosed channel.

## 2. Stokes flow and stokeslets

The Stokes flow equations describing the inertialess dynamics of an incompressible Newtonian fluid are

2.1 $$-\nabla p+\mu \nabla^{2}u=0$$

and

2.2 ∇⋅u=0,

where *p* = *p*(***x***, *t*) is pressure, ***u*** = ***u***(***x***, *t*) is velocity, ***x*** is position, *t* is time and the constant μ is dynamic viscosity. These equations are typically accompanied by the no-slip, no-penetration condition ***u***(***X***, *t*) = ∂_*t*_***X*** on boundary points ***X***, along with ***u*** → 0 or ***u*** ∼ ***U***_ambient_ as |***x***| → ∞ in exterior flows.

The dynamic geometric complexity characterizing many biological flows complicates both analytical and numerical solution approaches; however, the linearity of equations (2.2) mean that solutions can be constructed by superposing ‘fundamental’ solutions in the absence of a boundary driven by point forces, moments, sources/sinks and higher order derivatives. For example, Hancock’s [3] slender body theory was based on replacing the sperm flagellum with a line integral of point forces (which he termed *stokeslets*) combined with appropriately weighted source dipoles to account for the finite radius of the flagellum. Similarly, the flow due to a translating sphere in Stokes flow can be decomposed exactly as a point force and source dipole.

Another key feature of equations (2.2) is the absence of an explicit time derivative, which means that flow is instantaneously determined by the boundary conditions. This absence results in the famous scallop theorem [33], i.e. that a swimmer must undertake a time-irreversible motion in order to achieve a net translation (see also the earlier movie of Taylor [34]); moreover a microscopic swimmer cannot ‘kick and glide’; continuous motion requires continuous expenditure of energy. From a computational perspective, the absence of a time derivative entails that the flow problem itself does not require time-stepping, although the time-evolving transport of suspended particles or migration of swimmers of course does.

Focusing on fundamental solutions, the Stokes flow equations with a finite but spatially concentrated force located at ***y*** and pointing in the *k*-direction are

2.3 $$-\nabla p+\mu \nabla^{2}u+\delta (x-y){\hat{e}}_{k}=0$$

and

2.4 ∇⋅u=0,

where *δ*(***x***) is the three-dimensional Dirac delta distribution and ${\hat{e}}_{k}$ is a unit basis vector pointing in the *k*-direction. Equations (2.4) have solution ***u*** = (8*πμ*)^−1^(*S*_1*k*_, *S*_2*k*_, *S*_3*k*_) and *p* = (4*π*)^−1^*P*_*k*_, where

2.5 $$S_{jk}(x,y)=\frac{\delta_{jk}}{|x-y|}+\frac{\left(x_{j}-y_{j})(x_{k}-y_{k}\right)}{|x-y{|}^{3}}$$

and

2.6 $$P_{k}(x,y)=\frac{x_{k}-y_{k}}{|x-y{|}^{3}}.$$

This *Oseen tensor* [35] or *stokeslet* [3] forms the basis for slender body theory [3,4], along with the boundary integral method [36–38]. The boundary integral method enables the flow exterior to or bounded by a smooth surface *B* to be expressed as

2.7 $$u_{k}(y)=-\frac{1}{8\pi \mu }\iint_{B}S_{jk}(x,y)f_{j}(x)dS_{x}+\frac{1}{8\pi }\iint_{B}T_{jk\ell }(x,y)u_{j}(x)n_{\ell }(x)dS_{x},$$

where ***f*** is the traction, i.e. the force per unit area exerted by the fluid on the surface, ***n*** is the unit normal pointing into the fluid, and *T*_*jk*ℓ_ is the stress tensor −*P*_*k*_*δ*_*j*ℓ_ + μ(∂_ℓ_*S*_*jk*_ + ∂_*j*_ *S*_ℓ*k*_). The summation convention over repeated Cartesian indices is assumed above and throughout.

For problems satisfying $\iint_{B}u\cdot ndS_{y}=0$, i.e. no change in volume, the second integral term (‘double layer potential’) in equation (2.7) can be eliminated (see for example [39]), motivating focus on the single layer boundary integral equation

2.8 $$u_{k}(y)=-\frac{1}{8\pi \mu }\iint_{B}S_{jk}(x,y)f_{j}(x)dS_{x}.$$

Using the fact that *S*_*jk*_(***x***, ***y***) = *S*_*kj*_(***y***, ***x***) and relabelling, equation (2.8) may be rewritten [38]

2.9 $$u_{j}(x)=-\frac{1}{8\pi \mu }\iint_{B}S_{jk}(x,y)f_{k}(y)\;dS_{y}.$$

The well-known advantages of the boundary integral are (1) reducing the computational domain from a three-dimensional (3D) volume to a two-dimensional surface (or possibly one-dimensional line) *B*, and (2) the natural treatment of open boundaries without the need to make artificial truncations, enabling excellent computational accuracy and efficiency. For example, Phan-Thien *et al.* were able to model propulsion of a physiologically shaped bull sperm cell in the mid-1980s, making use of machines with the order of 1 MB memory [37].

The standard numerical approach to the solution of equation (2.9) is to discretize the traction as

**Figure 1.**
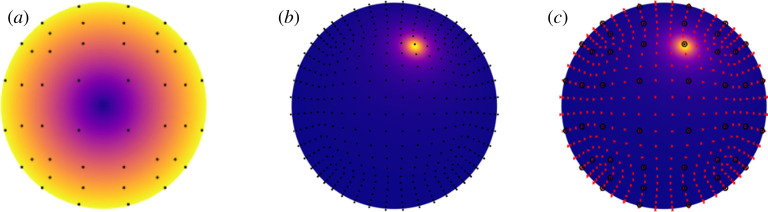
The problem of discretizing the regularized stokeslet boundary integral equations via quadrature rules, illustrated via the resistance problem for a rigid sphere, with associated coarse discretization illustrated via dots. (*a*) The traction field associated with pure rotation, with associated fine discretization illustrated via crosses. (*b*) The regularized stokeslet kernel with ε = 0.1 for fixed stokeslet point ***y*** and varying field point ***x***. (*c*) The combination of coarse (large black dot) and fine (red/light grey dot) discretizations as employed by the nearest-neighbour regularized stokeslet method. (Online version in colour.)

2.10 $$f(y)\approx \sum_{n=1}^{N}f[n]\phi_{n}(y),$$

where ***f***[1], …, ***f***[*N*] are vector constants and *ϕ*_1_(***y***), …, *ϕ*_*N*_(***y***) are basis functions (see figure 1 for a sketch of the discretization over a rigid sphere). In the simplest case, the latter may be piecewise constant functions defined on a partition $B=B_{1}\cup \ldots \cup B_{N}$ via *ϕ*_*n*_(***y***) = 1 for ***y*** ∈ *B*_*n*_ and *ϕ*_*n*_(***y***) = 0 otherwise. The semi-discrete numerical problem then reads

2.11 $$u_{j}(x)=-\frac{1}{8\pi \mu }\sum_{n=1}^{N}(\iint_{B_{n}}S_{jk}(x,y)dS_{y})f_{k}[n].$$

The resistance problem (prescribed velocity ***u*** on the surface, unknown traction ***f***) can be solved as follows: equation (2.11) can be converted to a 3*N* × 3*N* linear system by applying point collocation, i.e. ***x*** = ***x***[*m*] for *m* = 1, …, *N* where ***x***[*m*] is the centroid of element *B*_*m*_. As discussed below, the swimming problem is a minor modification of the resistance problem involving the introduction of an unknown rigid body motion and additional force and moment balance equations.

However, scientists who are not computational specialists may encounter two challenges in implementing the above approach.

(i) The generation of a *mesh*, i.e. the connected, non-overlapping partition *B*_1_, …, *B*_*N*_ of what may be a complex geometry. Typically this mesh includes a set of vertices, a connectivity table associating each element *B*_*n*_ to 3 or 4 vertices, a mapping between local coordinates (*ξ*, *η*) in each element and global coordinates ***y*** and surface metric *dS*_***y***_.
(ii) The evaluation of the (weakly) singular integrals occurring in equation (2.11) when ***x*** = ***x***[*m*] and *n* = *m*. These integrals involve terms which behave as *r*^−1^ as *r* → 0. A related difficulty occurs ‘downstream’ of the problem for the traction if subsequent computation of the velocity ***u***(***x***) at a fluid point ***x*** close to *B* is required.

## 3. Regularized stokeslets

Issues (i) and (ii) are certainly not insurmountable, and codes such as the Fortran library BEMLIB [40] provide considerable assistance. However, given the scientific breadth of (often experimentally-focused or multidisciplinary) researchers working in biological fluid dynamics, there is great value in methods which avoid or alleviate them. One appealing strategy is to *regularize* the singularity occurring so that ${\tilde{S}}_{jk}(x,x)<\infty$, which immediately solves issue (ii). Additionally issue (i) can then be side-stepped by simply covering *B* with a set of points {***x***[1], …, ***x***[*N*]} (without the need for a connectivity table or local coordinates), and discretizing the integral directly with a quadrature rule,

3.1 $$\iint_{B}{\tilde{S}}_{jk}(x,y)f_{k}(y)dS_{y}\approx \sum_{n=1}^{N}{\tilde{S}}_{jk}(x,x[n])F_{k}[n],$$

where *F*_*k*_[*n*]: = *f*_*k*_(***x***[*n*]) d*S*(***x***[*n*]), i.e. absorbing the quadrature weight and metric into the unknown force. The linear system for the resistance problem then becomes,

3.2 $$u_{j}(x[m])=-\frac{1}{8\pi \mu }\sum_{n=1}^{N}{\tilde{S}}_{jk}(x[m],x[n])F_{k}[n].$$

Equation (3.2) is referred to as a Nyström discretization of the integral equation [41].

There are clearly many ways to regularize the kernel, for example replacing |***x*** − ***y***|^−1^ with (|***x*** − ***y***|^2^ + ε^2^)^−1/2^ for some small parameter ε > 0. However, this ad hoc approach has the unwanted side-effect of producing potentially unphysical ‘solutions’ that no longer satisfy the original equations, for example by violating the incompressibility condition ∇⋅u=0. Cortez and colleagues [23,24] proposed instead to start by regularizing the point force, i.e. to consider the exact solution to the Stokes flow equations with spatially-smoothed point forces,

3.3 $$-\nabla p+\mu \nabla^{2}u+\phi_{\varepsilon }(x-y){\hat{e}}_{k}=0,$$

and

3.4 ∇⋅u=0,

where *ϕ*_ε_(***x***) is a family of “blob” functions which approximates *δ*(***x***) as ε → 0. The particular choice

**Figure 2.**
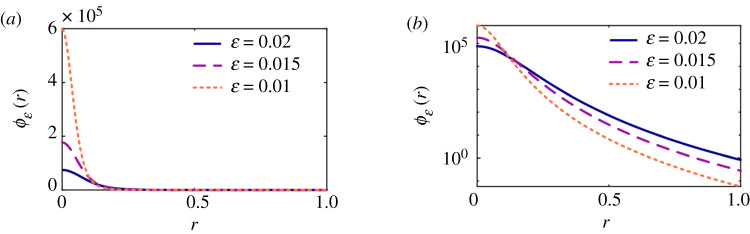
The spatially-smoothed approximation *ϕ*_ε_ to the 3D Dirac delta function of Cortez *et al.* [24], plotted in spherical polar coordinates, for values ε = 0.02, 0.015, 0.01 on (*a*) linear and (*b*) log-linear axes. (Online version in colour.)

3.5 $$\phi_{\varepsilon }(x)=\frac{15\varepsilon^{4}}{8\pi {\left(|x{|}^{2}+\varepsilon^{2}\right)}^{7/2}},$$

is plotted in figure 2. This choice leads to the regularized stokeslet solutions

3.6 $$P_{k}^{\varepsilon }(x,y)=\frac{\left(x_{k}-y_{k}\right)}{{\left(|x-y{|}^{2}+\varepsilon^{2}\right)}^{5/2}}(2|x-y{|}^{2}+5\varepsilon^{2})$$

and

3.7 $$S_{jk}^{\varepsilon }(x,y)=\frac{\delta_{jk}(|x-y{|}^{2}+2\varepsilon^{2})+(x_{j}-y_{j})(x_{k}-y_{k})}{{\left(|x-y{|}^{2}+\varepsilon^{2}\right)}^{3/2}}.$$

It can be seen that $P_{k}^{\epsilon }(x,y)∼P_{k}(x,y)$ and $S_{jk}^{\varepsilon }(x,y)∼S_{jk}(x,y)$ as ε → 0; moreover the corresponding single layer boundary integral equation is

3.8 $$u_{j}(x)=-\frac{1}{8\pi \mu }\iint_{B}S_{jk}^{\varepsilon }(x,y)f_{k}(y)dS_{y}+O(\varepsilon^{p}),$$

where *p* = 1 for ***x*** on or near *B* and *p* = 2 otherwise [24]. Alternative forms for the blob function have been derived [17] to improve the order of the error; however, the form (3.5) is by far the most commonly used. The *O*(ε) regularization error associated with boundary collocation is inherited by the resistance problem for finding the traction associated with a given rigid body motion, and the swimming problem for finding the traction and translation/rotation resulting from a given boundary deformation and force/moment balance. It is distinct from the errors associated with discretization of the traction and numerical quadrature which we will consider shortly.

Regularization enables a particularly convenient discretization of the single layer boundary integral equation; this simplicity however comes at a cost. The Nyström method (3.2) corresponds to using identical discretizations for the traction *f*_*k*_(***y***) and *S*_*jk*_(***x***, ***y***) when considered as functions of position ***y*** ∈ *B* (the latter for fixed ***x***), despite the fact that the stokeslet kernel varies much more rapidly than the traction, as shown in figure 1, the variability becoming more rapid as ε is reduced (figure 2). The number of degrees of freedom of the system 3*N* and hence the 3*N* × 3*N* matrix size is tied to the discretization for the traction, and so reducing the regularization error via reducing ε entails rapid growth in memory and computational requirements associated with system assembly and solution. Cortez *et al.* [24] initially suggested a quadrature error *O*(*h*^2^/ε^3^) where *h* is the discretization length; more recently [42] this estimate has been improved to *O*(*h*^2^/ε) + *O*(*P*ε^−1/*P*^ *h*^1−*P*^) for any integer *P* > 3.

As described in equations (2.10), (2.11), boundary element methods separate out the traction and quadrature discretization by expanding the traction in terms of basis functions, leaving the stokeslet integrals to be evaluated by the most appropriate means (e.g. adaptive quadrature) without affecting the number of degrees of freedom of the system. Alongside being the standard method for the classical boundary integral equation, this approach has been employed in the context of *regularized* stokeslets to model cilia-driven flow [43,44], autophoretic swimmers [45] and to explore the evolution of bacterial morphology [46]. These studies would have been challenging or practically impossible via the Nyström method, which computational experiments suggest would have required above 3 orders of magnitude greater memory and processing time [47]. A related idea of pre-integrating line distributions of regularized stokeslets to model cilia and flagella, which is closely related to slender body theory [47] has recently been extended to higher order basis functions [48,49], and to model flagellar elastohydrodynamics [50]. However, problems involving surface integrals require true mesh generation and as such despite being suggested over 10 years ago [47], the ‘element’ approach has not been very widely adopted in comparison with the Nyström method for regularized stokeslets.

With the aim of preserving the meshlessness of the Nyström discretization but decoupling the traction discretization from the numerical quadrature, we recently [51] suggested an alternative approach based on an idea from meshless interpolation. The concept is to use two point cloud discretizations, one ‘coarse force’ set {***x***[1], …, ***x***[*N*]} which is sufficient to capture the variation of the traction, and which dictates the size of the linear system, and another finer quadrature discretization set {***X***[1], …, ***X***[*Q*]} which has sufficient resolution to capture the rapidly varying kernel $S_{jk}^{\varepsilon }(x,y)$ for ***y*** in the vicinity of ***x***, as in figure 1*c*. The force at a quadrature point ***f***(***X***[*q*])*dS*(***X***[*q*]) is then approximated by its value at the nearest point on the coarse force set, $f(x[\hat{n}])dS(x[\hat{n}])$, where the index $\hat{n}$ is given by

3.9 $$\hat{n}=\underset{n=1,\ldots ,N}{\text{argmin}}|x(n)-X(q)|.$$

Denoting $\nu [q,\hat{n}]=1$ and *ν*[*q*, *n*] = 0 for $n\neq \hat{n}$, we may write

3.10 $$\begin{array}{rl} u_{j}(x[m]) & \approx -\frac{1}{8\pi \mu }\sum_{q=1}^{Q}S_{jk}^{\varepsilon }(x[m],X[q])f_{k}(X[q])dS(X[q]) \end{array}$$

3.11 $$\begin{array}{rl} & \approx -\frac{1}{8\pi \mu }\sum_{q=1}^{Q}S_{jk}^{\varepsilon }(x[m],X[q])\sum_{n=1}^{N}\nu [q,n]f_{k}(x[n])dS(x[n]) \end{array}$$

3.12 $$\begin{array}{rl} & =\frac{1}{8\pi \mu }\sum_{n=1}^{N}\left(\sum_{q=1}^{Q}S_{jk}^{\varepsilon }(x[m],X[q])\nu [q,n]\right)F_{k}[n], \end{array}$$

where *F*_*k*_[*n*]: = −*f*_*k*_(***x***[*n*])*dS*(***x***[*n*]).

Defining *h*_*f*_ to be the length scale associated with the traction points and *h*_*q*_ to be the length scale associated with the quadrature points, equation (3.12) has a regularization error *O*(ε) and traction discretization error *O*(*h*_*f*_). The quadrature error depends on whether the traction points are ‘contained’ (i.e. within distance *O*(ε) or closer) in the quadrature set. Associated to each traction point which is contained in the quadrature set is an error $O(\varepsilon^{-1}h_{q}^{2})+O(P\varepsilon^{-1/P}h_{q}^{1-1/P})$ for all integer *P* > 3, the former term being dominant [42]. Associated to each traction point which is no closer than distance *δ* ≫ ε > 0 to the quadrature points is an error $O(h_{q}^{3}\delta^{-2})+O(Ph_{q}^{1-2/P})$ [42]. In the latter case there is no asymptotic dependence on ε in the limit ε → 0.

The ‘NEAREST’ method (3.12) is not intended to compete directly with boundary element methods, still less techniques based on treecodes and fast multipole methods [52–54], rather it is aimed at providing a simple and accessible meshfree method for non-specialists that is capable of dealing with problems of moderate complexity, with relatively standard workstation hardware. As an example, the resistance matrix (forces and moments due to the translation and rotation of a sphere), with regularization parameter ε = 0.01 can be calculated to within 2% error with *N* = 864 and *Q* = 3456, taking 6 seconds on a notebook computer. The same computation with the Nyström method (*N* = *Q* = 3456) yielded a 5% error and required 350 seconds. Other early applications of the nearest-neighbour discretization by our group have included the diffusion tensor of a macromolecular structure [51], cell motility [31] and embryonic nodal flow [32].

A further feature of this method is that equation (3.12) can be viewed as the product of a (dense) 3*N* × 3*Q* stokeslet matrix with a 3*Q* × 3*N* (sparse) nearest-neighbour matrix and a 3*N* × 1 column vector of tractions, which naturally lends itself to implementation in numerical linear algebra software such as MATLAB or GNU Octave in terms of matrix–matrix and matrix–vector operations. Limited use of an iterated loop to handle the ‘Q’ dimension in block prevents memory overflow in in this respect. The use of ‘under the hood’ numerical linear algebra enables ongoing progress in hardware and software optimizations—particularly those involving multicore and graphical processing unit (GPU) developments—to be exploited ‘passively’, i.e. with minimal changes to the code. Careful investigation of the effect of parallelization is warranted; algorithms may differ by orders of magnitude in their performance on parallel architectures, and on GPU hardware in particular. It is important to note that inherently serial algorithms will yield at best no improvements on parallel hardware, and at worst can exhibit worse performance. We conclude this review by presenting some experiments along these lines; it will be found that the use of a modest compute-GPU in constructing and solving swimming-type problems can lead to a reduction in the required computational time that can be in excess of an order of magnitude when using the nearest-neighbour regularized stokeslet method.

## 4. Parallelizing NEAREST

In a recent article [31], we showed how the trajectories of multiple flagellated swimmers with specified beat patterns can be calculated through formulating and solving an initial-value problem (IVP) of the form $\dot{Y}=F(Y,t)$, where

$$Y(t)=\left[\begin{array}{c} X_{0}(t)\\ B(t) \end{array}\right],$$

with **X**_0_(*t*) being the position of the swimmers at time *t*, and **B**(*t*) the matrix of basis vectors describing the body-frame of the swimmers. The rates ${\dot{X}}_{0}(t)$ and $\dot{B}(t)$ are determined from the linear system for the swimmers’ translational velocity **U** (3 scalar unknowns per swimmer), angular velocity **Ω** (3 scalar unknowns per swimmer) and tractions **f**[ · ] (3*N* scalar unknowns per swimmer). This formulation allows for the use of built-in IVP solvers such as Matlab’s ode45, or GNU Octave’s lsode. Approximately 90–99% of the computational time needed to solve such a problem using NEAREST is due to a combination, at each time step, of

(i) construction of the stokeslet matrix multiplied by the nearest-neighbour matrix (*A*_*s*_), such as in (3.12);
(ii) construction of the matrix (*A*) from pre-calculated matrix blocks;
(iii) solution of the linear system.

The construction of the matrix *A*_*s*_ can itself be decomposed into two steps: the calculation of the regularized stokeslet kernel at each force node with respect to each quadrature node (3*N M* × 3*Q M* calculations for *M* swimmers), and, then, the multiplying of the stokeslet matrix with the nearest-neighbour matrix *ν*[*q*, *n*]. This is exactly the type of problem that is suited to GPU optimization; a large number of small calculations can take advantage of the large number of processing cores available on modern GPUs, with relatively modest hardware enabling thousands of calculations to be performed simultaneously. Similarly, it is well known that there are significant gains to be made when using a GPU for large matrix construction [55], and with the NEAREST method we are able to exploit commercially optimized algorithms for solving linear systems on the GPU, such as the Matlab “\” command.

With the aim of maintaining the simplicity of the original method we take a *passively parallel* approach whereby we do not rewrite the entire method to fully optimize GPU use, but make minimal changes that nonetheless bring an order of magnitude performance improvement. For detailed description of the previously published code and algorithms, see refs [31,51]; the entirety of the code changes to make use of an available compute GPU are as follows.

(i) To construct the matrix *A*_*s*_, the vectors containing force and quadrature points (*x* and *X* respectively) are ‘cast’ to the GPU via the commands
  x=gpuArray(x),X=gpuArray(X).
  There is a small amount of computational overhead in transferring data between the CPU and GPU, but many Matlab operators are ‘overloaded’ to work on both standard and GPU arrays without additional intervention by the user. Owing to the use of *x* and *X* as gpuArray s, the constructed matrix *A*_*s*_ is itself a gpuArray, and therefore the large matrix *A* is constructed on the GPU, again with no additional user intervention.
(ii) The Matlab “\” command for solving the linear system works without modification on a gpuArray, outputting a gpuArray solution vector $\dot{F}$. Currently the Matlab built-in ODE solvers (such as ode45) are not currently GPU optimized, and so the solution must be ‘gathered’ from the GPU back to the CPU via the command
  $$\dot{F}=gather(\dot{F}).$$

In what follows we will investigate the effect that these minimal user changes can have on the run time of simulations, including an assessment of the savings for each of the three major costs (assembling *A*_*s*_, assembling *A*, linear solver), and how these changes impact the time required to solve ‘real-world’ problems based on previous work of tracking nine sperm swimming between two solid plate boundaries (an extension of [31]), and of tracking particle deposition in the *euciliated* embryonic node of the mouse. Once we have established how the use of GPU processing can improve existing computations, we will apply these modifications to two new sets of simulations involving (1) an array of 25 individually modelled swimming *Caenorhabditis elegans*, and (2) the problem of particle transport by an array of sperm swimming between two solid plate boundaries.

### (a) Benchmarking

To benchmark the improvements when using a GPU to accelerate NEAREST, we make use of three machines:

(**M1**) Lenovo Thinkstation, with 2x Intel Xeon 4116 CPUs (each with 12 cores), 128 GB DDR4 RAM, and an NVIDIA Quadro RTX 5000 GPU (3072 parallel-processing cores, 16 GB GDDR6 RAM);
(**M2**) Lenovo NeXtScale server *BEAR HPC* (Birmingham Environment for Academic Research High Performance Computing) processing unit (CPU), using 1x Intel Xeon E5-2640 (10 cores) and 64 GB DDR4 RAM;
(**M3**) Lenovo NeXtScale server *BEAR HPC (GPU)*, using 1x Intel Xeon E5-2640 CPU (10 cores), 64 GB DDR4 RAM, and an NVIDIA Tesla P100 GPU (3584 parallel-processing cores, 12 GB HBM2 RAM).

Comparisons will be made between calculations on M1 (with and without GPU acceleration), and between M2 (CPU) and M3 (GPU). Although M2 and M3 form part of an HPC cluster, each calculation will be limited to a single computational node per simulation.

For benchmarking we take the swimming problem of a single model sperm from [31]. The model sperm comprises a tail of length *L* = 50 μm, discretized as a line of stokeslets, and an ellipsoidal head with semi-axes of length 2 μm, 1.6 μm and 1 μm, and is non-dimensionalized with respect to the length-scale *L*. The beat pattern follows the planar activated beat of Dresdner & Katz [56]. We discretize the model sperm tail using *N*_*t*_ = 100 and *Q*_*t*_ = 400, and an increasing number of traction points on the head *N*_*h*_ with *Q*_*h*_ = 4*N*_*h*_, to obtain a problem in terms of 3(*N*_*h*_ + 100) scalar d.f., and corresponding 3(4*N*_*h*_ + 400) quadrature points.

**Figure 3.**
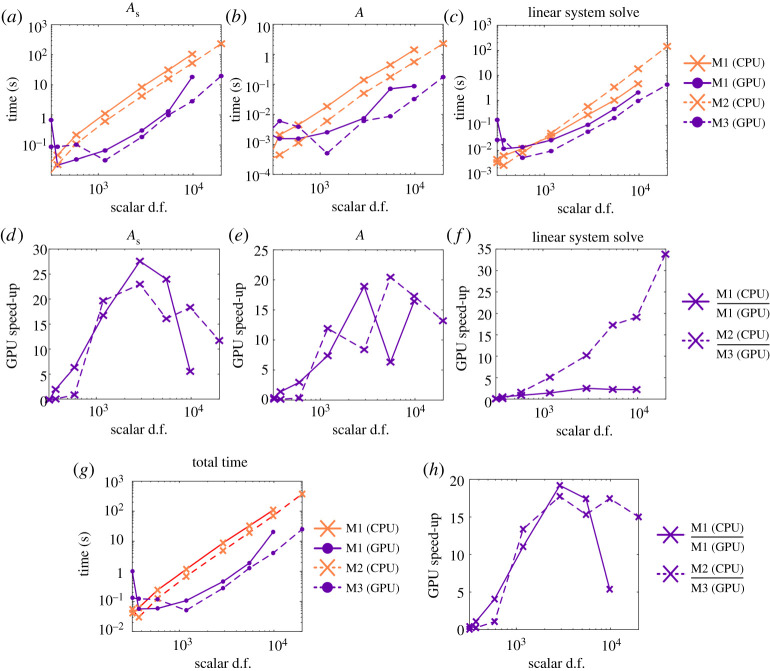
Comparison of the computational time for constructing the matrices *A*_*s*_ and *A*, and solving the linear system for the problem of a single sperm swimming. (*a*–*c*) The time taken for M1 (CPU), M1 (GPU), M2 and M3 (lower is better). (*d*–*f* ) The relative speed increase when incorporating the GPU for M1, and when using M3 instead of M2 (higher is better). (*g*) The total time to solve a single iteration for the single sperm on M1 (CPU), M1 (GPU) (lower is better), M2 and M3. (*h*) The relative speed increase when incorporating the GPU for M1 and M3 instead of M2 (higher is better). (Online version in colour.)

In figure 3 we show the time required for calculation of the model swimming sperm at the first time point *t* = 0. In these figures, the times shown are the mean time calculated over 100 runs. Figure 3*a*–*c* shows the total time taken for constructing *A*_*s*_, *A* and for solving the linear system, with the relative increases (M1 (CPU))/(M1 (GPU)) and M2/M3 shown in figure 3*d*–*f* . In each case we see that for small numbers of scalar d.f. (<500) the CPU calculations show a modest performance increase over those with GPU acceleration. When increasing the number of d.f., we see greater than an order of magnitude speed up for *A*_*s*_ (maximum gains are a factor of 29 (M1) and 23 (M2/M3)), *A* (maximum gains are a factor of 19 (M1) and 20 (M2/M3)), and for solving the linear system on M2/M3 (34 times faster). When solving the linear system on M1 for >500 scalar d.f., we see a factor of 2 increase when using the GPU as opposed to the CPU. This reduced increase is due to the availability of 24 CPU cores on M1, allowing for significant performance increases over M2. For very large numbers of scalar d.f. (>10^4^) there is a slight reduction in computational gains when using the GPU machines. This is due to the limited amount of GPU memory available, meaning additional overhead in transferring data between the CPU and GPU. Figure 3*g*,*h* shows the total time taken to construct and solve the swimming problem for the first time step *t* = 0. Here same pattern occurs, with an order of magnitude speed up when using the GPU for problems with >10^3^ scalar d.f., with a maximum 21 times increase for M1, and 18 times increase for M2/M3.

### (b) Multiple swimming sperm between parallel plates

**Figure 4.**
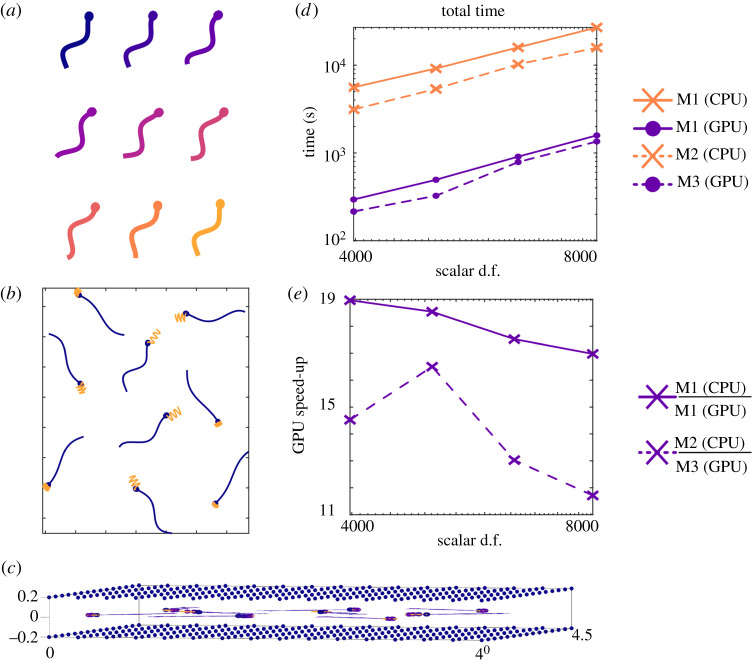
Simulation of an array of 9 model sperm for three beat cycles. (*a*) The model beat pattern in time. (*b*) The position of each swimmer at *t* = 0 is shown, with the paths traced out by the leading point as they swim overlain. (*c*) Side-on view of swimmers showing location of plate boundaries. (*d*) The total simulation time required on each of M1 (CPU), M2 (GPU), M3 (CPU) and M4 (GPU) (lower is better). (*e*) The relative speed when using a GPU compared to CPU on M1, and M3 compared to M2 (higher is better). (Online version in colour.)

In the first of our ‘real-world’ tests, we assess the performance of the GPU parallelized method for a set of nine sperm swimming between two parallel plates, using the knowledge that regularized stokeslet methods enable solid boundaries to be taken into account via the inclusion of additional surface distributions. In the present case we consider the situation of two parallel plane walls approximating a microscope slide and coverslip. Between these, we simulate an array of nine model sperm, again using the model of Dresdner & Katz [56], initialized with varying wavenumber *k* ∈ [2*π*, 4*π*] and phase *ϕ* ∈ [0, 2*π*], and solved for three beat cycles. Boundaries are included as a pair of parallel rectangular plates 4 × 4.5 flagellar lengths (equivalently 180 × 202.5 μm), separated by a distance of 0.4 flagellar lengths (equivalently 18 μm), with the sperm swimming in the plane a distance of 0.2 flagellar lengths from each plate. The characteristic beat pattern of one of these swimmers is shown in figure 4*a*, with the position of each swimmer at time *t* = 0, and the tracks traced out over three beat cycles shown in figure 4*b*. The boundaries (as shown in figure 4*c*) are discretized with a total of 1440 scalar d.f. (and corresponding 7680 vector quadrature points), with the sperm tails discretized using 120 scalar d.f. (and corresponding 160 vector quadrature points), and the heads are discretized with an increasing number of scalar d.f. (162–648 per swimmer). For the range of scalar d.f. shown in figure 4*d*,*e*, we see in excess of an order of magnitude speed increase when using the GPU accelerated machines, with a maximal 19 × speed-up when using the GPU on M1 with 3978 scalar d.f.

### (c) Particle tracking in the *euciliated* mouse node

The flow driven by beating cilia in a fluid-filled structure called the embryonic node is the initiator of symmetry breaking in early development [30,43,44], although the mechanism by which this flow is converted into anatomical asymmetry is still to be understood [22]. The role of morphogen-bearing vesicles is believed to play a central role.

To investigate this problem, we recently modelled the enclosed embryonic node of the mouse using NEAREST [32], with a biologically realistic 112 beating cilia, with particular focus on how the calculated flow can transport particles potentially needed for chemical signalling. The large number of beating cilia, and non-planar boundaries in this problem, means that long time scale computational simulation of particle transport is particularly challenging for most existing methods.

**Figure 5.**
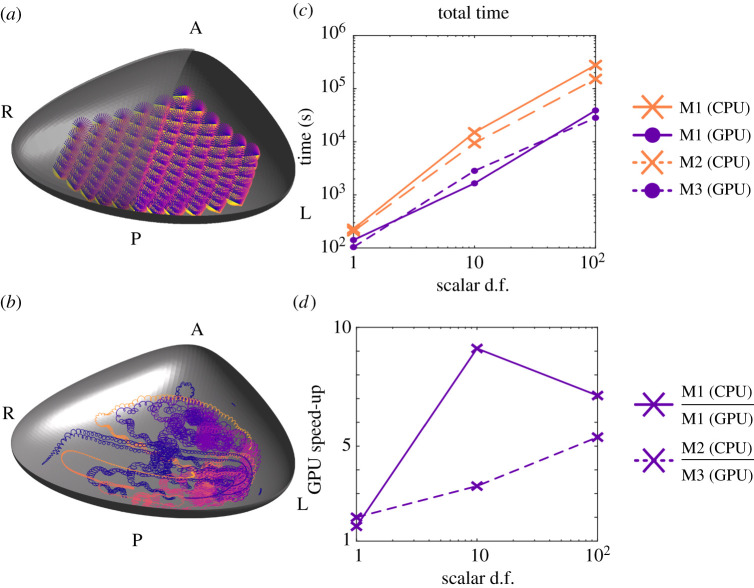
Particle tracking in the *euciliated* mouse node. (*a*) A sketch of the mouse node geometry with overlaying Reichert’s membrane and 112 beating cilia. (*b*) Paths traced out by 10 particles after 1000 beats. (*c*) The total simulation time required on each of M1 (CPU), M2 (GPU), M3 (CPU) and M4 (GPU) (lower is better). (*d*) The relative speed when using a GPU compared to CPU on M1, and M3 compared to M2 (higher is better). (Online version in colour.)

Taking the forces calculated for the 39 979 scalar d.f. from the previous work [32], we calculate the time required to track a set of 1, 10 and 100 particles for 1000 beat cycles of the 112 cilia. A sketch of the *euciliated* mouse node is shown in figure 5*a*, with the paths of 10 particles shown in figure 5*b*, time requirements in figure 5*c* and relative speed increase in figure 5*d*. We see in the tracks the characteristic leftward-moving, ‘loopy drift’ of particles as they follow the flow generated by the array of beating cilia. The ability to model and perturb a physiologically accurate node, with particle transport over a long time, alongside the mechanical stresses produced by cilia movement, may enable us to probe more deeply the flow conversion mechanism that has proved to be so elusive, and moreover to assess the diverse morphologies observed in different species.

For the GPU accelerated calculations, the size of the system requires significant data transfer between the GPU and the CPU, and so the relative speed increases are not as significant—up to almost an order of magnitude on M1, and half an order of magnitude when using M3 rather than M2. However the real-world implication of this speed increase is significant; when solving for a system of 100 particles, the time required is a little over 77 hours when using M1 (CPU), but less than 11 hours on M1 (GPU).

## 5. Parallelization enables simulation of large problems

We have shown that the inclusion of GPU parallelization enables significant speed up for computations using the NEAREST method, both in individual benchmarks (§4a) and when comparing to ‘real-world’ calculations (§4b,c). We will now highlight how this method can be used to investigate other problems in computational biology, such how fluid mixing and transport can occur due to the presence of suspensions of microswimmers [57–59]. Specifically, we will focus on the simulation of a large array of *C. elegans* swimmers, and the question of whether the small, rapidly decaying, velocity disturbance caused by swimming sperm is sufficient for particle transport.

### (a) Multiple swimming *Caenorhabditis elegans*

**Figure 6.**
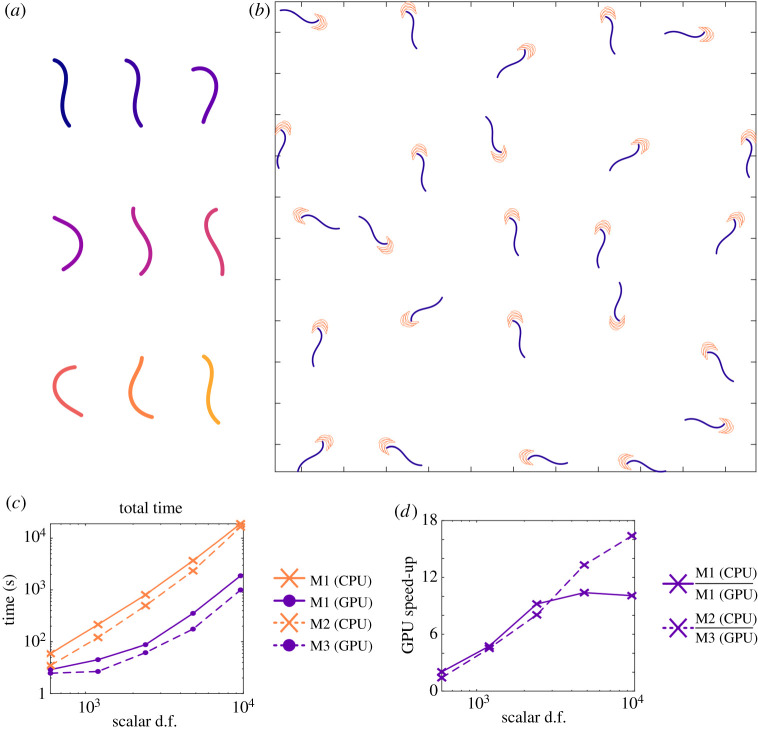
Simulation of an array of 25 model *C. elegans* for three beat cycles. (*a*) The model beat pattern in time. (*b*) The position of each swimmer at *t* = 0 is shown, with the paths traced out by the leading point as they swim overlain. (*c*) The total simulation time required on each of M1 (CPU), M2 (GPU), M3 and M4 (lower is better). (*d*) The relative speed when using a GPU compared to CPU on M1 and M3 compared to M2 (higher is better). (Online version in colour.)

We simulate an array of 25 model *C. elegans* over three beat cycles (figure 6*b*). Each swimmer is discretized as a line of stokeslets with 75*N* scalar d.f., and corresponding *Q* = 25 × 4*N* vector quadrature points. The *C. elegans* beat pattern follows that of Thomases & Guy [60], illustrated for a single swimmer in figure 6*a*. The time taken to solve this swimming problem when *N* = 2^*n*^ (*n* = 3, 4, …, 7) is shown in figure 6*c*, with the speed up when using the GPU on M1, or using M3 instead of M2, shown in figure 6*d*. As with the single iteration calculations in §4a, when using a reasonable number of points (>96 scalar d.f. per swimmer) we see an order of magnitude speed up in calculations. When using 384 scalar d.f. to discretize each swimmer, the real-world time required is just over 4.5 h when using the CPU of M2, reducing down to around 15 min on the GPU of M3.

### (b) Particle transport by sperm swimming between boundaries

**Figure 7.**
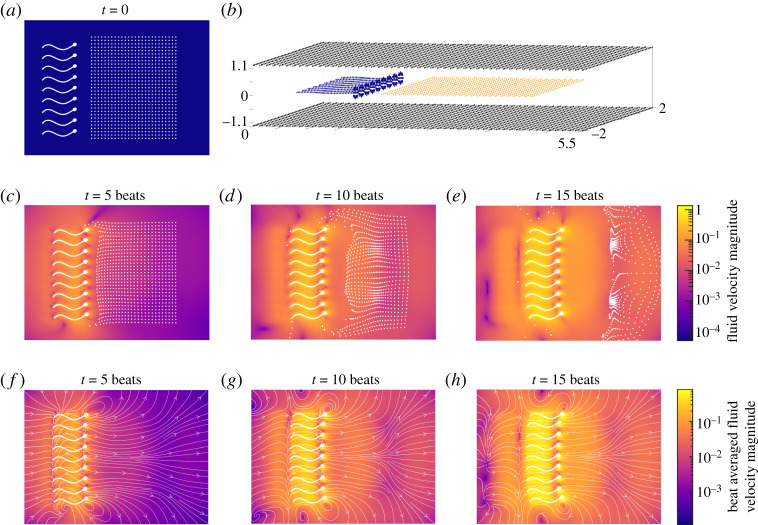
Simulation of particle transport by a line of 9 model sperm between two flat plates over 15 beat cycles. (*a*) The position of the cells and particles at time *t* = 0. (*b*) Sketch of the set-up showing the cells between two flat plates (note the axes are presented with non-equal scaling, causing the cell heads to appear more circular in this plot than they are in simulations). (*c*–*e*) The position of the cells and particles at intervals of 5 beat cycles, plotted over the velocity magnitude of the fluid disturbance. (*f* –*h*) The position of the cells at intervals of 5 beat cycles, plotted over the beat-averaged velocity magnitude of the fluid disturbance with accompanying ‘instantaneous’ streamlines. In panels (*c*–*h*) velocities are scaled with respect to the flagellar length multiplied by the beat frequency, with the colour shown on a logarithmic scale. (Online version in colour.)

The GPU implementation enables several aspects of interest to be combined to obtain results that may not have previously been possible. In this, final, example we investigate the particle transport by sperm swimming between two flat parallel plates. In this simulation we initialize nine individually modelled sperm, placed in a line aligned half way between two parallel plates, with the centroid of each cell separated by a distance of 9 *r*_2_ (with *r*_2_ being the half-width of the cell head). The rigid boundaries are rectangular with dimensions 5.5 × 4 flagellar lengths, and are placed a dimensional distance of 10 μm apart to mirror those the common clinical research set-up of a shallow glass imaging chamber. An array of 26 × 31 particles, separated in each direction by 0.1 flagellar lengths, is placed 0.5 flagellar lengths ahead of the sperm. In these simulations the boundaries comprise 15606 scalar d.f., with 2538 scalar d.f. for the cells, and 2418 scalar d.f. for the particles. A sketch of the cells and particles can be seen as a top-down view in figure 7*a*, and of the cells, particles, and boundaries in figure 7*b*.

The results of these simulations after 15 beats are shown in figure 7. In Figure 7*c*–*e*, the position of the cells and particles is shown, together with the magnitude of the fluid velocity disturbance (shown on a logarithmic scale). Additionally in Figure 7*f* –*h*, we show the beat-averaged fluid velocity magnitude, together with ‘instantaneous’ streamlines. A counterintuitive finding is that these small instantaneous velocities resulting from the rapid decay of the flow field around the swimmers (and even smaller beat-averaged velocities) are nevertheless sufficient to result in transport that, initially at least, has a larger average velocity than the progressive speed of the individual sperm cells driving the flow. The ability to transport particles ahead of the sperm may serve as part of an important biological process; could this provide evidence for a potential mechanism by which a population of sperm can introduce chemical messengers from semen into the cervix and uterus? Further experimental work would be required to confirm this, however the results in figure 7 suggest it is at least hydrodynamically feasible.

The fluid dynamics of transport in narrow films or between plates is of longstanding interest to the biofluids community. Liron & Mochon [14] showed that the flow generated by a stokeslet between two parallel flat plates (later extended to thin films by Mathijssen *et al.* [61]) exhibits source-dipole-like far-field behaviour, decaying as 1/*r*^3^. We would hence expect the flow generated by a swimmer (or set of swimmers) to decay like 1/*r*^4^; an observation which is consistent with the results in figure 7, in which the transported particles are most densely located approximately 1 flagellar length away in front of the swimming cells, with the rapid decay causing the particles to bunch together. The problem of particle mixing in such confined flows is also important for understanding many biological processes. Pushkin & Yeomans [62] demonstrated how domain confinement significantly changes the transport of particles by swimming cells and its dependence on swimmer kinematics. To complement analysis based on statistical description and idealized far fields, parallel simulation approaches such as those described here should enable more specific details of cell morphology, beat kinematics and geometry/complexity of the confining environment to be taken into account.

The results presented here suggest that GPU parallelization can enable flow fields and particle disturbance in confined geometries to be precisely resolved; moreover the computational framework should also enable swimming and transport in complex and dynamic environments such as the female reproductive tract to be computed.

## 6. Discussion

The regularized stokeslet method provides a relatively elementary access point to numerical simulation of the geometrically complex Stokes flows characterizing many microscale biological systems. In this paper we reviewed the nearest-neighbour discretization approach, which decouples the number of degrees of freedom from the quadrature process, hence controlling the size of the linear system that needs to be solved. We then described the implementation of this method on GPU-enabled hardware; the fact that the method is already based on linear algebra operations means that only two lines of Matlab code needed to be added in order to exploit GPU acceleration. Assessing the method on existing problems of calculating swimming motion due to multiple sperm in a confined channel, and particle transport in a ciliated organ, as well as new problems involving multiple undulatory swimmers in an infinite fluid, we consistently observed *at least* an order of magnitude time reduction when using the GPU over the CPU. We have also demonstrated the versatility of this method by assessing the problem of predicting particle transport by multiple sperm swimming in a directed fashion between two parallel plates. While we carried out some of the computations on an HPC cluster, we limited our use to a single computational node per simulation. Further advances may be possible by re-analysing the code and/or using a compiler, although our primary focus is simplicity of implementation.

Parallel computing methods for microscale flow have been explored since the development of the completed double layer boundary integral equation method of Phan-Thien and Tullock in 1994 [63] (see also the earlier work of Power & Miranda [64] and later by Keaveny & Shelley [65]), enabling a multiparticle system with 81 000 degrees of freedom to be solved on a Cray-YMP class supercomputer. The most powerful approaches to large scale microscale flow problems are based on approximating the multipole expansion of the stokeslet, including the kernel-independent fast multipole method [52,53,66,67], treecode [54] and the force-coupling method [68,69], which enable *O*(*N*) or *O*(*N*log *N*) computational complexity, by contrast with the *O*(*N*^3^) complexity of the method described here. These ideas have been used to simulate large suspensions of swimmers and elastic fibres, which are beyond the scope of the ‘original’ formulations of the boundary integral method or regularized stokeslet method, arguably at the cost of significantly greater implementational complexity. More ‘mainstream’ computational fluid dynamics approaches based on volumetric meshes and the finite volume and finite element methods have also been deployed very successfully for multicilia simulations for example [70]. It would certainly be interesting to compare the present code with these methods, however the spirit of the nearest-neighbour approach is, rather than aiming to reduce asymptotic complexity, to minimize unnecessary degrees of freedom (i.e. to reduce *N*), via algorithms that can be implemented in a few linear algebra operations, and without intricate mesh construction. Future efforts along these lines may consider how rapidly-advancing algorithmic and hardware developments can continue to be made available to the non-specialist community.

## Data accessibility

Matlab code to calculate the results and create the figures in this paper can be found at https://gitlab.com/meuriggallagher/passively-parallel-regularized-stokeslets, and the GPU accelerated NEAREST package can be found at https://gitlab.com/meuriggallagher/nearest
